# Molecular and Clinical Profiles of Human Pegivirus Type 1 Infection in Individuals Living with HIV-1 in the Extreme South of Brazil

**DOI:** 10.1155/2019/8048670

**Published:** 2019-06-11

**Authors:** Luísa D. Da Mota, Fabiana Finger-Jardim, Cláudio M. Silva, Fabiana N. Germano, Maiba M. Nader, Carla V. Gonçalves, Karen Y. Sánchez Luquez, José A. B. Chies, Andrea V. Groll, Vanusa P. Da Hora, Jussara Silveira, Rossana P. Basso, Marcelo A. Soares, Ana M. B. Martínez

**Affiliations:** ^1^Molecular Biology Laboratory, School of Medicine, Universidade Federal do Rio Grande, Rio Grande, Rio Grande do Sul, Brazil; ^2^Molecular Biology Laboratory, School of Medicine, Universidade Federal Fluminense, Rio de Janeiro, Brazil; ^3^Laboratory of Immunogenetics, Bioscience Institute, Universidade Federal do Rio Grande do Sul, Porto Alegre, Rio Grande do Sul, Brazil; ^4^Oncovirology Program, Brazilian National Cancer Institute (INCA), Rio de Janeiro, Brazil

## Abstract

Human pegivirus type 1 (HPgV-1) infection has been associated with a beneficial effect on the prognosis of human immunodeficiency virus type 1 (HIV-1)-coinfected individuals. However, the mechanisms involved in this protection are not yet fully elucidated. To date, circulating HPgV-1 genotypes in HIV-1-infected individuals have not yet been identified in the extreme south of Brazil. The present study aimed to determine the genotypic circulation of HPgV-1 and the influence of HPgV-1 status and persistence time on the evolution of HIV-1 infection. A retrospective cohort of 110 coinfected individuals was analyzed. Samples were subjected to viral RNA extraction, cDNA synthesis, nested PCR, and genotyping. Genotypes 1 (2.8%), 2 (47.9% of subtype 2a and 42.3% of subtype 2b), and 3 (7%) were identified. In antiretroviral treatment-naïve subjects HPgV-1 subtype 2b was associated with lower HIV-1 viral load (VL) rates (*p* = 0.04) and higher CD4+ T-cell counts (*p* = 0.03) than was subtype 2a, and the positivity for HPgV-1 was associated with higher CD4+ T-cell counts (*p* = 0.02). However, there was no significant difference in HIV-1 VL between HPgV-1-positive and HPgV-1-negative subjects (*p* = 0.08). There was no significant association between the different groups in HPgV-1 persistence and median HIV-1 VL (*p* = 0.66) or CD4+ T-cell counts (*p* = 0.15). HPgV-1 subtype 2b is associated with better prognosis of HIV-1 infection. Although HPgV-1 infection is persistent, our data suggest that the time of infection does not influence HIV-1 VL or CD4+ T-cell counts in coinfected subjects.

## 1. Introduction

Human pegivirus type 1 (HPgV-1), formerly named GB virus type C (GBV-C), is an RNA virus of the Flaviviridae family and of the* Pegivirus* genus [1]. To date, seven HPgV-1 genotypes have been described which are distributed in different geographic regions [2–4]. Genotype 1 predominates in West Africa; genotype 2, in Europe, USA, South America, and North Africa; genotype 3, in Asian and Amerindian populations; genotype 4, in Southeast Asia and the Philippines; genotype 5, in South Africa; genotype 6, in Indonesia; and genotype 7, in China [3, 4]. HPgV-1 is found mainly in natural killer (NK) cells, T and B lymphocytes, and monocytes [5]. HPgV-1 is transmitted by parenteral, sexual, or maternal-infant routes [6–9]. The prevalence of HPgV-1 viremia in healthy blood donors ranges from 1% to 19% [10, 11].

Most studies involving HPgV-1 are related to its coinfection with HIV-1 [12–15]. The presence of HPgV-1 RNA appears to be associated with increased survival and lower mortality of HIV-1-positive patients [12, 14]. In addition, HPgV-1 coinfection of hepatitis C virus (HCV) and HCV/HIV-1 carriers appears to decrease the severity of liver disease, and, in those coinfected with the Ebola virus, survival appears to be higher than that in monoinfected individuals [15, 16].

The mechanism by which HPgV-1 influences the progression of certain viral infections has not yet been fully elucidated. One of the hypotheses suggests that the presence of HPgV-1 reduces T-cell activation by modulating the immune system to better perform against viral agents [5, 17]. In addition, it has been suggested that this immune response occurs only in the presence of certain HPgV-1 genotypes [18, 19]. On the other hand, HPgV-1 has also recently been negatively associated with other diseases, such as non-Hodgkin's lymphoma [20, 21], multiple sclerosis [22], and severe encephalitis [23].

The present study aimed to determine the genotypic circulation of HPgV-1 and the influence of HPgV-1 status and persistence time on the evolution of HIV-1 infection in a cohort of HIV-1/HPgV-1 coinfected patients followed up at a reference center in the southernmost part of Brazil.

## 2. Materials and Methods

### 2.1. Study Design and Population

This is a retrospective cohort study that evaluated the HPgV-1/HIV-1 coinfection of 110 patients, all of whom were followed up at the Infectious Diseases Department of Dr. Miguel Riet Corrêa Jr. University Hospital (HU-FURG) in the city of Rio Grande, RS, in the extreme south of Brazil. Sociodemographic, economic, and behavioral information was obtained from the study database previously assembled [6]. Clinical and laboratory data were obtained after review of medical records. The evolution of HIV-1 infection was estimated through HIV viral load and CD4+ T-cell count trajectories. Plasma samples were collected by the Laboratory of HIV Viral Load and CD4+ T-cell counts (BioLab) of HU-FURG between 2002 and 2016. An aliquot of each sample was stored in the BioLab Biorepository at −70°C and made available for the present study. The protocol of this study has been approved by the Ethics and Health Research Committee (CEPAS) of the Federal University of Rio Grande (FURG) (#103/2012).

### 2.2. Inclusion Criteria

**Figure 1:**
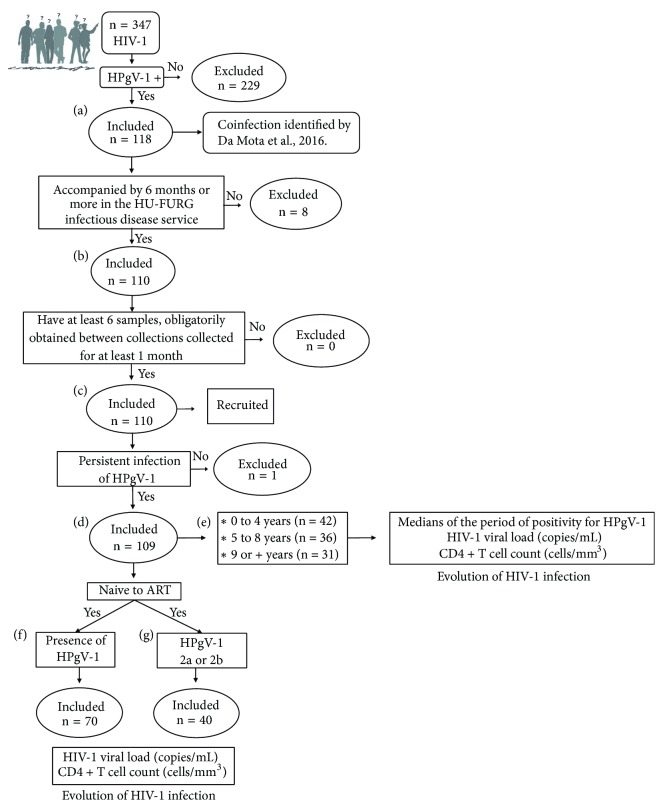
Study design. (a) HPgV-1/HIV-1-coinfected population identified by Da Mota et al. [6]. (b) Coinfected followed up by the HU-FURG infectious disease service for a minimum of 6 months. (c) Individuals with at least 6 biological samples available, obtained at intervals of at least 1 month. (d) HPgV-1 persistence time. (e) Time of HPgV-1 persistence in 3 categories. Influence of HPgV-1 persistence on the evolution of HIV-1 infection. (f) Effect of the presence of HPgV-1 on the progression of HIV-1 infection in antiretroviral-naive subjects. (g) Influence of HPgV-1 genotypes 2a and 2b on the evolution of HIV-1 infection in antiretroviral treatment-naïve subjects.

Initially, we included HPgV-1/HIV-1 coinfected individuals (n = 118) (Figure 1(a)). Of those, we selected subjects followed for at least six months at HU-FURG (n = 110) (Figure 1(b)). In addition, those subjects had at least six biological samples available at BioLab, obtained through collections at intervals of at least one month (n = 110) (Figure 1(c)). All enrolled patients were tested for hepatitises B (HBsAg) and C (anti-HCV). These individuals were recruited to HU-FURG and invited to participate in the study. All subjects signed a free and informed consent form.

To assess whether persistence time of HPgV-1 infection modifies the evolution of HIV-1 infection, individuals with persistent HPgV-1 infection were selected (n = 109) (Figure 1(d)). A persistent infection was defined if the virus was detected for more than six months. This definition was based on the time criterion for establishing chronicity for HCV [24]. The time period evaluated in each patient was at least 6 months, varying further according to the availability of the samples. The persistence time was grouped into 3 categories (Figure 1(e)) to allow a more robust comparison between the groups. For each patient, the median values of HIV-1 viral load and CD4+ T cell counts, corresponding to the positivity period for HPgV-1, were estimated.

To assess the effect of HPgV-1 and its subtypes 2a and 2b on the progression of HIV-1 infection, subjects who were naive to antiretroviral therapy (ART) were studied to assess the patients' natural history. Of the 109 patients recruited to evaluate HPgV-1 persistence, 70 had samples without the influence of ART (Figure 1(f)), and, of those, 40 comprised subtype 2a (n = 19) or 2b (n = 21) (Figure 1(g)). HIV-1 viral load, CD4+ T cell counts, and HPgV subtypes were obtained from the same sample tested for HPgV-1.

### 2.3. Pilot Study

**Figure 2:**
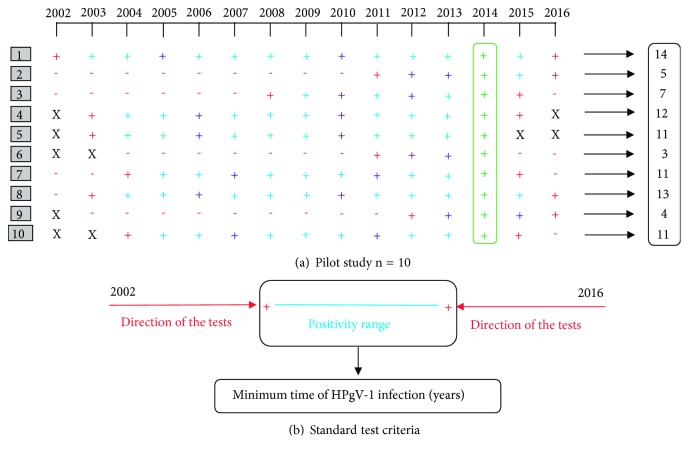
Laboratory results of the pilot study. (a) Each line (1 to 10) corresponds to the laboratory results of a patient's HPgV-1 test. The year of sample collection is represented in columns. Molecular HPgV-1 positivity is indicated by the + symbol and negativity by -. The red crosses indicate samples that have been tested until finding the positivity range. Purple crosses indicate samples that have been tested as internal quality controls of the positivity range. Blue crosses indicate samples that have not been tested, because the positivity interval had already been estimated. Green crosses indicate samples that have been tested in a previous study that identified HPgV-1/HIV-1 coinfections [11]. The X's indicate unavailable samples. (b) The graph illustrates the standard criterion established for the test. The red arrows indicate the time direction of the samples to be tested until the positivity range indicated by the blue line is determined.

A pilot study was performed with approximately 10% (n = 10) of the initial population (10/110). Subjects were randomly selected, and each had one sample per year (2002 to 2016) tested for HPgV-1 RNA positivity. A pattern was observed in the molecular behavior of HPgV-1 since only one period of positivity occurred in each individual, with no indication of reinfection (Figure 2(a)).

Following these observations, we chose to define a standard protocol for subsequent HPgV-1 molecular tests. It was established that we would start by testing the oldest (closest to 2002) and the most recent (closest to 2016) sample of each participant. If a given result was negative, the test would proceed to the following (or previous) sample, according to the directions shown by the red arrows in Figure 2(a). This direction was followed until finding the HPgV-1 positive interval for each subject (blue line in Figure 2(b)). This period was considered the time of HPgV-1infection per subject. By establishing this methodology, it was also considered that other studies identified only one period of infection by HPgV-1 [8, 13, 25, 26]. For quality control of the established standard testing strategy, two samples contained in the HPgV-1 positivity range were also processed per patient. In addition, every 10 patients had a representative sample per year tested. Figure 2(b) shows the results of the pilot study. Their different colors indicate which samples would have been tested if the standard test criteria were used for them.

### 2.4. Molecular Tests

#### 2.4.1. Viral RNA Extraction and cDNA Synthesis

Viral RNA was extracted from 140 *μ*L of plasma using the QIAamp Viral RNA Extraction Kit (QIAGEN), according to the manufacturer's protocol. Ten microliters of the extracted RNA was added to 300 ng of random oligonucleotides (2 *μ*L of 150 ng/solution, N6, Life Technologies, Itapevi, Brazil) and denatured at 70°C for 10 minutes. For cDNA synthesis, 200 U of Superscript reverse transcriptase (Thermo Fisher Scientific, Pittsburgh, PA), 0.1 M DTT, 5 U of RNaseOUT® (Life Technologies, Forster City, CA), and 0.5 mM of each deoxynucleotide were added. The cDNA reaction was conducted at 42°C for 1.5h in a final volume of 20 *μ*L.

#### 2.4.2. Nested-PCR for HPgV-1 Detection

After obtaining the cDNA, a nested polymerase chain reaction (nested-PCR) was used to detect HPgV-1. The virus noncoding genomic region (5′NCR) was amplified by adapting a PCR protocol described by Jarvis* et al*. [27]. The first round was performed with 5 *μ*L of the obtained cDNA and the second with 5 *μ*L of the product of the first PCR. Both reactions used 1x PCR Buffer, 2 mM MgCl_2_, 0.5 mM dNTPs, 1U of recombinant Taq DNA polymerase enzyme (Invitrogen, Carlsbad, CA), Milli-Q water q.s.p. to a final volume of 50 *μ*L, and 0.5 *μ*M of primers HGV1 and HGV2 (first round) or HGV3 and HGV4 (second round). Primer sequences were as follows: HGV1 forward 5′-AGGTGGTGGATGGGTGAT3′; HGV2 reverse 5′-TGCCACCCGCCCTCACCCGAA-3′; HGV3 forward 5′-TGGTAGGTCGTAAATCCCGGT-3′; HGV4 reverse 5′- GGAGCTGGGTGGCCCCATGCAT-3′. PCRs were performed in a thermocycler with the following cycling: initial denaturation at 95°C for 10 min, followed by 40 cycles of 94°C for 30 sec, 55°C for 30 sec, and 72°C for 30 sec, and a final extension stage at 72°C for 2 min. PCR products from the second round were electrophoresed on a 1.5% agarose gel. For this, 5 *μ*L of the PCR product from each sample was mixed with 1 *μ*L of Blue Green Loading Dye (LGC Biotecnologia, São Paulo, Brazil). Gels were visualized on a UV transilluminator and images were captured. A positive control for HPgV-1 (providing a PCR band of 344 bp), confirmed by direct sequencing of the PCR product, and an HPgV-1-negative sample were used, in addition to a blank reaction containing no DNA sample.

#### 2.4.3. HPgV-1 Genotyping and Phylogenetic Analysis

For the genotyping and phylogenetic analysis, PCR products were purified with the GFX PCR DNA and TM-Gel Band Purification kit (GE Healthcare, São Paulo, Brazil) and subjected to sequencing using the BigDye™ ABI PRISM 1 kit (Life Technologies). Afterwards, samples were sequenced on an ABI 3130xl Genetic Analyzer (Life Technologies) and the chromatograms obtained were edited manually in the SeqMan program (DNASTAR, Madison, WI). Sequence alignment was performed using the CLUSTALW algorithm implemented in the BioEdit package [28]. The obtained sequences were converted to FASTA format and were aligned with sequences representative of each HPgV-1 genotype. From the alignment, sequences were subjected to phylogenetic analysis by the neighbor-joining distance method (NJ). This algorithm provided the corrected genetic distances based on the evolutionary model of Kimura two-parameter (K2P). The confidence test of the generated topology was determined by the calculation of bootstrap values and those greater than 75% were considered significant. A total of 1000 replicates were performed in MEGA 7. The NJ method of phylogenetic reconstruction was chosen because of the greater computational speed. All sequences generated in this study were submitted to GenBank and were assigned the access numbers MH782477-MH782547.

#### 2.4.4. Statistical Analysis

Sociodemographic, behavioral, clinical, and laboratorial variables were analyzed in the statistical software SPSS for Windows v. 21 (IBM Corp., Armonk, NY). The population studied, the positivity for HPgV-1 in ART-naïve samples, the minimum time of HPgV-1 persistence, and the virus circulating genotypes were described using proportions and means. The prevalence of the HPgV-1 and subtypes 2a and 2b in the ART-naïve subjects were compared in relation to the HIV-1 VL and CD4+ T-cell counts with the* Mann-Whitney* U-Test. The low frequency of HPgV-1 genotypes 1 and 3 prevented their inclusion in the statistical analyses. In order to analyze the influence of HPgV-1 persistence time on the evolution of HIV-1 infection, three categories were created: 0 to 4 years (n = 42), 5 to 8 years (n = 36), and 9 or more years (n = 31). In this case, the analysis of variance (ANOVA) and the* Tukey* test were used.* p*-values ≤ 0.05 were considered statistically significant.

## 3. Results

### 3.1. General Characteristics of the Study Population

The initial population studied was composed of 110 patients coinfected with HPgV-1/HIV-1. A summary of the sociodemographic and clinical characteristics of the study population can be seen in Table 1. Seventy-five subjects (68.2%) self-declared to be white, 61 (55.5%) were male, 79 (71.8%) were single or without a fixed partner, the mean age was 40.4 years (SD ± 10.4), the average schooling was 7 years (SD ± 3.9), and average monthly income was R$ 1,007.89 (SD ± 885.40). Among the behavioral variables, 97 (88.2%) never used injecting drugs, 67 (60.9%) never used inhaled drugs, 71 (64.5%) had no tattoos, 85 (77.3%) never received transfusion blood, and the average number of partners was 2.6 per year (SD ± 4.1). Regarding clinical and laboratory variables, 91 never had hepatitis, and, among the 19 who had declared previous hepatitis, HCV was the known triggering agent in 10 and HBV in one. The minimum time of HIV-1 diagnosis was on average 8.2 years. The HIV-1 subtypes identified were subtype B (10%), subtype C (25.5%), subtype F1 (1.8%), and recombinant forms (13.6%). The remainder (49.1%) had no previously documented HIV-1 subtyping done.

### 3.2. Molecular Tests

A total of 797 samples were submitted to molecular testing by PCR-nested for HPgV-1. A total of 130 tests were performed during the pilot study and 292 after the pilot study, following the standard strategy established for the tests. Two hundred samples were tested for internal quality control of the positivity range; 105, for quality control for every 10 patients; and 70 tests for the samples from ART-naïve subjects.

### 3.3. HPgV-1 Genotype Circulation and the Influence on HIV-1 Infection

**Figure 3:**
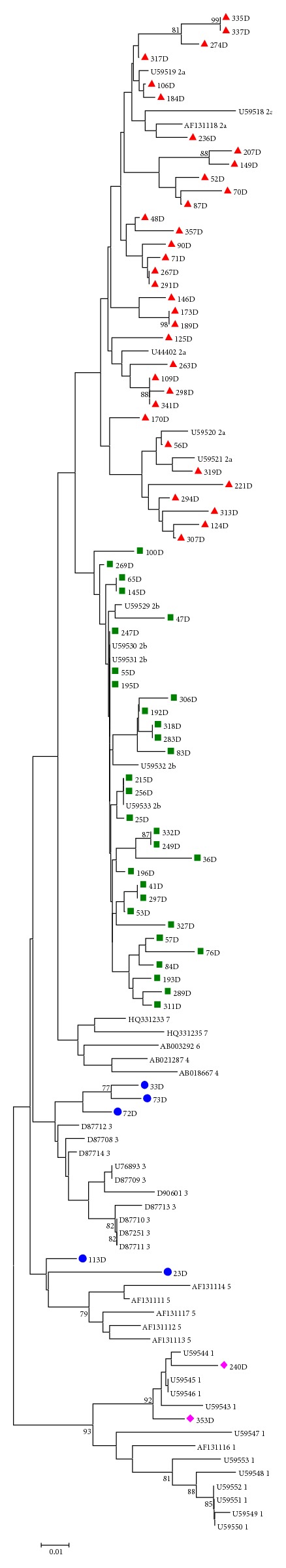
Phylogenetic tree of the circulating HPgV-1 genotypes in HIV-1-infected individuals in the extreme south of Brazil. Trees are based on sequences obtained from the 5′-NCR region of HPgV-1. An alignment with reference sequences of HPgV-1 genotypes 1 to 7 was performed. The isolates of the patients in this study are marked with diamonds (genotype 1), triangles (genotype 2a), squares (genotype 2b), or circles (genotype 3). References were obtained from the GenBank database and are identified by their accession number followed by the HPgV-1 genotype assigned thereto. Only bootstrap values above 75% are shown in the figure. The scale bar below the tree indicates 0.01 nucleotide substitutions per site.

**Figure 4:**
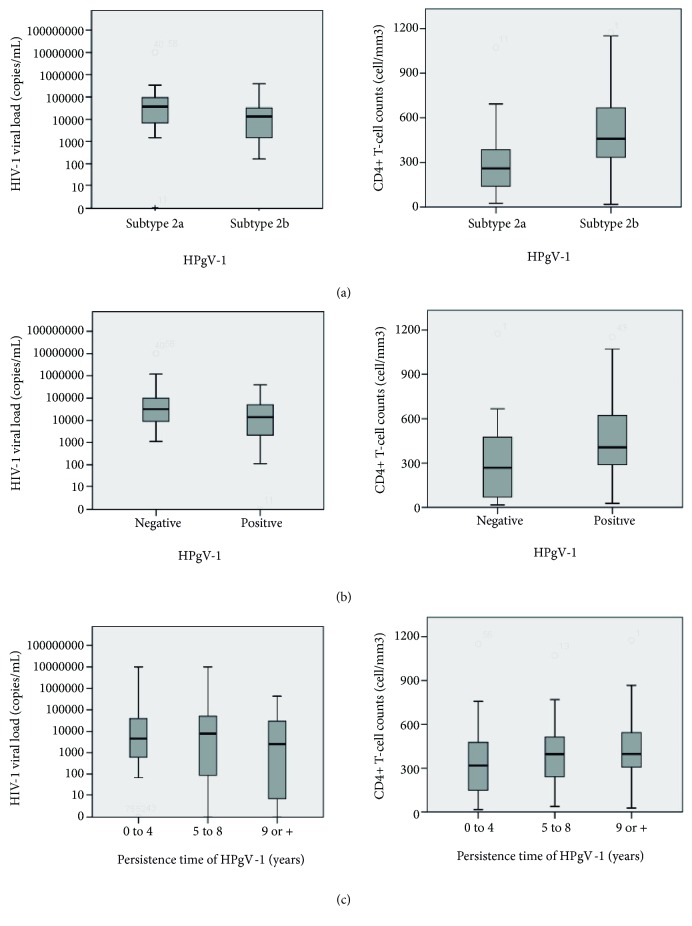
Box plots of the distribution of HIV-1 viral load median values (log scale) and CD4+ T-cell counts in HIV-1-infected individuals (linear scale). (a) Median values of the variables between HPgV-1 subtypes 2a and 2b (ART-naive subjects). (b) Median values of the variables between HPgV-1-positive and HPgV-1-negative (ART-naive subjects). (c) Median values of the variables among the different groups of HPgV-1 minimum persistence time in years: 0 to 4 years, 5 to 8 years, and 9 or more years. The differences between groups in (a) and (b) were calculated using the Mann-Whitney* U*-Test. Differences between groups in (c) were calculated using the analysis of variance (ANOVA).

HPgV-1 genotypes were determined in 71 (64.5%) of the 110 subjects studied. For the remaining 39 subjects, sequence quality was not adequate for phylogenetic analysis. Genotypes 1 (2; 2.8%), 2 (64; 90.1%), and 3 (5; 7%) were found. With respect to genotype 2, 34 (47.9%) were of subtype 2a and 30 (42.3%) were of subtype 2b.  Figure 3 illustrates the phylogenetic tree containing the genotypic circulation of the HPgV-1 in the HIV-1 positive population of southerner most Brazil. We observed that individuals infected with HPgV-1 subtype 2b had higher CD4+ T-cell counts (*Mann-Whitney* U-Test,* p* = 0.03) and lower HIV-1 VL (*Mann-Whitney *U-Test,* p* = 0.04) relative to subtype 2a (Table 2).  Figure 4(a) shows the distribution of HIV-1 VL and CD4+ T-cell counts between subjects infected with HPgV-1 subtypes 2a and 2b.

### 3.4. HPgV-1 in ART-Naïve Individuals and the Evolution of HIV-1 Infection

Of the 110 subjects recruited, 70 had samples available while being still ART-naïve. Of these, 40 (57.1%) were positive and 30 (42.9%) were negative for HPgV-1. Molecular positivity was significantly associated with higher CD4+ T-cell counts (Mann-Whitney* U*-Test,* p* = 0.02). However, there was no significant difference between groups with respect to mean HIV-1 VL (Mann-Whitney* U*-Test,* p* = 0.08) (Table 2).  Figure 4(b) shows the distribution of the HIV-1 VL and CD4+ T-cell counts between HPgV-1-positive and negative ART-naïve subjects.

### 3.5. Time of HPgV-1 Infection and Evolution of HIV-1 Infection

Of the 110 study participants, 109 had a persistent infection, all with a period over 1 year and a mean of 5.93 years (SD ± 3.54). There was no significant difference in the mean HIV-1 VL (ANOVA,* p* = 0.66) and CD4+ T-cell counts (ANOVA,* p* = 0.15) among the different groups of HPgV-1 persistence time.  Figure 4(c) depicts the distribution of HIV-1 VL and CD4+ T-cell counts between the different groups.

## 4. Discussion

The present study was the first to determine the genotypic circulation of HPgV-1 in HIV-1-positive individuals at an HIV/AIDS reference center in the extreme south of Brazil. In addition, the HPgV-1 persistence time and its influence on the evolution of HIV-1 infection were estimated.

The results of the phylogenetic analysis revealed the predominance of genotype 2 (subtypes 2a and 2b) in the population studied, while genotypes 1 and 3 were less frequently observed. These findings were similar to those found in other regions of Brazil, which detected the same genotypes in the HIV-1-infected population [10, 29, 30]. Another important observation in this study is that the five individuals coinfected with genotype 3 were infected with HIV-1 subtype C. In addition, in these individuals, HIV-1 infection preceded HPgV-1 infection. Thus, the possibility of HIV-1 subtype C strains acting as a facilitating agent to HPgV-1 genotype 3 infection should be further evaluated. With respect to genotype 2, subtype 2b was associated with an improvement in the prognosis of HIV-1 patients compared to subtype 2a. The median HIV-1 viral load in those infected with HPgV-1 subtype 2b was significantly lower, as were the higher CD4+ T-cell counts. The fact that some HPgV-1 strains influence the clinical course of HIV-1 infection more than others has already been suggested [19, 31–33]. Subtype 2b and genotype 5 were already related to higher rates of CD4+ T-cells compared to subtype 2a and genotype 1 [19, 33], and genotype 7 has already been associated with slower progression to AIDS [34]. It is thought that the different HPgV-1 genotypes have different tropisms to the CXCR4 and CCR5 coreceptors used by HIV-1 for entry into host cells, which would be determinant in the evolution of HIV-1 infection [35]. The competition for these coreceptors has also been suggested [36], since both viruses present cell tropism to CD4+ T-cells [37]. Blocking of these coreceptors was also proposed, which would be dependent on the production of cytokines stimulated by HPgV-1 viremia [38, 39].

The present study demonstrated that HPgV-1 establishes a persistent infection in individuals living with HIV-1. Some studies have associated persistent viremia of HPgV-1 with an improvement in the evolution of HIV-1 infection [12, 40]. However, the present study found that persistence does not appear to influence the evolution of HIV-1 infection, since there was no significant difference between median HIV-1 VL and CD4+ T-cell counts in the different groups of HPgV-1 persistence time.

Antiretroviral-naïve HPgV-1/HIV-1-coinfected subjects had higher CD4+ T-cell counts compared to HIV-1-monoinfected individuals. However, after ART initiation, this study did not observe an association between HPgV-1 and the median HIV-1 VL or CD4+ T-cell counts. Some studies have indicated that HPgV-1 may act to reduce T-cell activation [41–43], benefiting coinfected individuals even after the introduction of therapy, with a likely synergy between both viruses [15, 42]. The beneficial effects of HPgV-1 have already been associated with other viral infections, such as those with HCV [44] and Ebola [16]. Studying the effects of this viral agent in different populations and understanding its mechanisms of action may be useful in developing novel antiviral therapies.

## 5. Conclusions

The present study showed that human pegivirus type 1 is a persistent infection in individuals living with HIV-1. It is suggested that the active infection influences the CD4+ T-cell counts of ART-naïve individuals, which could explain the improvement in the prognosis of HIV-1-infected individuals. The data from the present study also suggest that HPgV-1 subtype 2b may be directly related to a better prognosis. In addition, HIV-1 subtype C strains may act as facilitators of genotype 3 HPgV-1 infection. However, further studies are needed to clarify the mechanisms that HPgV-1 uses during infection, whether related to host or viral factors.

## Figures and Tables

**Table 1:** Sociodemographic and clinical characteristics of the studied population, FURG (2002-2016).

Variable/category	N (%)	Mean (± SD)
*Skin color* ^*a*^		
Non-white	35 (31.8)	-
White	75 (68.2)	-
*Gender*	-	-
Female	49 (44.5)	-
Male	61 (55.5)	-
*Age (yr) *	-	40.4 (±10.4)
*Schooling (yr)*	-	**7 **(±3.9)
*Monthly income* ^*b*^	-	1,007.89 (±885.4)
*Marital status*		
Married / with a fixed partner	31 (28.2)	-
Single / without a fixed partner	79 (71.8)	-
*Number of sexual partners *	-	2.6 (±4.1)
*Injecting drug user*		
No	97 (88.2)	-
Yes	13 (11.8)	-
*Inhaled drug user*		
No	67 (60.9)	-
Yes	43 (39.1)	-
*Tattoo*		
No	71 (64.5)	-
Yes	39 (35.5)	-
*Blood transfusion*		
No	85 (77.3)	-
Yes	25 (22.7)	-
*Hepatitis C virus (anti-HCV+)*		
No	100 (90.9)	-
Yes	10 (9.1)	-
*Hepatitis B virus (HBsAg+) *		
No	109 (99.1)	-
Yes	1 (0.9)	-
*Time since HIV-1 diagnosis* ^*a*^		8.2 (±5.2)
*HIV-1 infecting subtype *		
B	11 (10.0)	-
C	28 (25.5)	-
F1	2 (1.8)	-
Recombinants forms	15 (13.6)	-
ND	54 (49.1)	-

SD, standard deviation; ND, not done.

^a^According to the classification of the Brazilian Institute of Geography and Statistics.

^b^Income in Brazilian reais (R$).

**Table 2:** Analysis of the mean HIV-1 VL and CD4+ T-cell counts stratified by HPgV-1 subtypes 2a and 2b and HPgV-1 molecular status among ART-naïve subjects.

*People living with HIV-1*	*Variables*				
		HIV-1 VL		CD4+ cell counts	
		(copies/ml)		(cells/mm^3^)	
	N	Mean	*p∗*	Mean	*p∗*
		rank		rank	
HPgV-1 subtype 2a	19	24	**0.04** ^#^	16	**0.03**
HPgV-1 subtype 2b	21	16		24	

HPgV-1 positive	40	30	0.08	41	**0.02**
HPgV-1 negative	30	40		29	

*∗*Mann-Whitney *U*-Test. Mean ranks.

^#^Statistically significant results are indicated in bold.

## Data Availability

The data used to support the findings of this study are available from the corresponding author upon request.

## Additional Points

*Study Limitations.* The present study suffers from some important limitations. First, due to the low sample size of HPgV-1 genotypes 1 and 3 present in our study, statistical analysis associations with coinfecting HIV-1 subtype or with modified HIV-1 disease course could not be performed with all HPgV-1 genotypes found. Also, we cannot exclude the possibility that our lack of association between time of HPgV-1 persistence and HIV laboratory markers of disease progression was due to small number of patients when breaking them down into three groups. Studies with larger cohorts may be able to tease out such effect. Finally, we were not able to measure cytokine/chemokine and their receptor levels, which are suggested to explain the effect of HPgV-1 coinfection in HIV+ subjects.
